# Investigation of Mitochondrial Metabolic Response to Doxorubicin in Prostate Cancer Cells: An NADH, FAD and Tryptophan FLIM Assay

**DOI:** 10.1038/s41598-017-10856-3

**Published:** 2017-09-05

**Authors:** Shagufta Rehman Alam, Horst Wallrabe, Zdenek Svindrych, Ajay K. Chaudhary, Kathryn G. Christopher, Dhyan Chandra, Ammasi Periasamy

**Affiliations:** 1 The W.M. Keck Center for Cellular Imaging, Physical and Life Sciences Building, University of Virginia, 90 Geldard Dr., Charlottesville, Virginia 22904 USA; 2 Roswell Park Cancer Institute, Centre for Genetics and Pharmacology, Department of Pharmacology and Therapeutics, Elm & Carlton Streets, Buffalo, New York 14263 USA; 3 Departments of Biology and Biomedical Engineering, University of Virginia, 90 Geldard Dr., Charlottesville, Virginia 22904 USA

## Abstract

Prostate cancer (PCa) is one of the leading cancers in men in the USA. Lack of experimental tools that predict therapy response is one of the limitations of current therapeutic regimens. Mitochondrial dysfunctions including defective oxidative phosphorylation (OXPHOS) in cancer inhibit apoptosis by modulating ROS production and cellular signaling. Thus, correction of mitochondrial dysfunction and induction of apoptosis are promising strategies in cancer treatment. We have used Fluorescence Lifetime Imaging Microscopy (FLIM) to quantify mitochondrial metabolic response in PCa cells by tracking auto-fluorescent NAD(P)H, FAD and tryptophan (Trp) lifetimes and their enzyme-bound fractions as markers, before and after treatment with anti-cancer drug doxorubicin. A 3-channel FLIM assay and quantitative analysis of these markers for cellular metabolism show in response to doxorubicin, NAD(P)H mean fluorescence lifetime (τ_m_) and enzyme-bound (a_2_%) fraction increased, FAD enzyme-bound (a_1_%) fraction was decreased, NAD(P)H-a_2_%/FAD-a_1_% FLIM-based redox ratio and ROS increased, followed by induction of apoptosis. For the first time, a FRET assay in PCa cells shows Trp-quenching due to Trp-NAD(P)H interactions, correlating energy transfer efficiencies (E%) vs NAD(P)H-a_2_%/FAD-a_1_% as sensitive parameters in predicting drug response. Applying this FLIM assay as early predictor of drug response would meet one of the important goals in cancer treatment.

## Introduction

Fluorescence Lifetime Imaging Microscopy (FLIM) is a powerful tool to assess the metabolic state of cells and tissues under different pathophysiological conditions using the auto-fluorescent properties of metabolic co-enzymes NAD(P)H and FAD^1, 2^. Both are predominantly located in the mitochondrial tricarboxylic acid cycle (TCA) and electron transfer chain (ETC) – FAD exclusively – producing ATP and reactive oxygen species (ROS) as part of the apoptosis pathway; NAD(P)H is also present in the cytosolic glycolysis pathway, important for highly proliferating cells like cancer^1, 3, 4^. The glycolysis pathway in the cytosol releases NADH and contributes to the free NADH pool. Mitochondrial oxidative phosphorylation (OXPHOS) activity consumes NADH (increased NADH-enzyme-bound fraction) and produces FAD (diminished FAD enzyme-bound fraction). Both the co-enzymes in their reduced (NAD(P)H and FADH_2_) and oxidized (NAD(P)^+^ and FAD) forms participate in the cellular oxidation-reduction reactions critical for cell physiology. Since the fluorescence spectra of NADH and NADPH cannot be readily distinguished^5^, we have chosen to use NAD(P)H through this manuscript.

The fluorescence lifetimes of NAD(P)H and FAD are sensitive to changes in pH, temperature, their conformational state and proximity to quenchers^6^. These co-enzymes exist in “free” or “enzyme-bound” states during cellular metabolic activity. FLIM is a very sensitive tool which allows discriminating the lifetimes and their relative fractions of free and enzyme-bound states of the co-enzymes from their fluorescence lifetime decay curve. Typically, fitting of lifetime decays of NAD(P)H and FAD are based on a two-component exponential decay model^6^. The shorter (0.4 ns) and longer (2.4 ns) lifetimes of NAD(P)H represent the “free” and “enzyme-bound” components, respectively. On the other hand, the shorter (0.12 ns) and longer (3.38 ns) lifetimes of FAD represent the “enzyme-bound” and “free” components, respectively. As mentioned above, NAD(P)H auto-fluorescence signals come from the cytosol and mitochondria whereas, FAD signals mostly originate from the mitochondria thus, both are regarded as reporters of metabolic activity^1, 3, 7^; their ratios are used as a marker of cellular redox states^8^. In this manuscript, enzyme-bound fractions of NAD(P)H (a_2_%) and FAD (a_1_%) and their lifetimes (τ_m_) are used to quantify these metabolic changes, in particular with our novel NAD(P)H-a_2_%/FAD-a_1_% FLIM-based redox ratio. The commonly used intensity-based redox ratio [NAD(P)H/FAD], based predominantly on signals driven by mitochondrial OXPHOS is defined as a reduction of this ratio, due to the conversion of fluorescent NAD(P)H to non-fluorescent NAD^+^ and conversion of non-fluorescent FADH_2_ to fluorescent FAD. We have instead used the above FLIM-based NADH-a_2_%/FAD-a_1_% ratio to avoid potential intensity-related artefacts (due to photo-bleaching, fluctuations in illumination sources, etc.) as a sensitive indicator of mitochondrial redox state. Here, an increase in metabolic activity is defined as an increase of this ratio, due to the increase of the fluorescent NAD(P)H enzyme-bound lifetime fraction a_2_% and decrease of the fluorescent FAD enzyme-bound fraction a_1_%^9^.

We are also introducing a new assay for the auto-fluorescent biomarker, tryptophan (Trp), which has been linked to cancer investigations^10–14^. Trp, an essential amino acid, is a precursor of niacin, which in turn is a precursor of NAD(P)H^15^. Increased Trp catabolism and increased indoleamine 2,3-dioxygenase (IDO) activity in the kynurenine pathway are linked to cancer development and progression^16^. Therefore, probing Trp is clinically relevant. In general, Trp fluorescence intensity and lifetime mainly provide information on the protein composition, protein structure of which they are a part of and changes in overall cellular microenvironment. Trp is therefore, used as a marker for protein abundance. Like NAD(P)H and FAD, Trp also has shorter (0.5 ns–2.5 ns) and longer (3.1 ns) lifetimes which represent the “protein-bound” (as residues in proteins) and “free” (free amino acid) lifetime components, respectively. One of the major applications of FLIM is the measurement of Förster resonance energy transfer or FRET^17–21^. In FLIM-FRET measurements, FRET events are identified if there is reduction in the donor lifetime, because of quenching of its fluorescence in the presence of the acceptor. Trp-NAD(P)H is a known FRET pair^6, 13, 22, 23^. The NAD(P)H-interacting enzymes carrying Trp residues from different metabolic pathways are potentially responsible for the quenching of Trp resulting in FRET. The efficiency of energy transfer or E% is calculated from the following equation E = 1 − τ_m_/τ_0_, where τ_m_ is the mean lifetime of Trp in cells and τ_0_ = 3.1 ns of unquenched Trp lifetime measured from Trp in solution. We have demonstrated that Trp-NAD(P)H interactions can be used as a reporter of metabolic activity^13, 23^.

In cancer there is re-programming of cellular metabolism. Mitochondrial OXPHOS is largely defective and cellular energy demands are met through a hyperactive glycolytic pathway, referred to as the Warburg Effect^24^. This characteristic shift in cancer cell metabolism has been linked with cell proliferation, progression and metastasis^24–26^. Mutations, deletions and reductions in mtDNA have been linked to defective OXPHOS activity, dysregulated production of ROS and hyperactive glycolytic pathway^27^. In PCa, reduced mtDNA and mitochondrial dysfunction have been demonstrated^27–29^. It is observed that African-American (AA) respond poorly to therapy as compared with Caucasian-American (CA) PCa patients. Although, the reason for this disparity could be multi-factorial, reduced mtDNA and mitochondrial dysfunction has been identified as the underlying cause^28^. Also, reduced mtDNA and defective OXPHOS have been linked with the resistance and/or attenuation to apoptosis in cancer cells^30, 31^. Therefore, induction of cell death by correction of impaired mitochondrial OXPHOS activity is a promising strategy in cancer treatment. Doxorubicin is one of the potent anti-cancer drugs known to induce apoptosis^32, 33^. In a recent study, it was shown that doxorubicin targets OXPHOS for apoptosis induction^29^. In the study, mitochondrial response to 10 µM doxorubicin treatment for 24 hr in colon and PCa cells showed increase in mitochondrial ROS, increase in cytochrome c release, increase in caspase-3 activation and increase in the percentage of apoptotic cells. All these markers show that there is induction of the mitochondrial apoptotic pathway eventually leading to cancer cell death.

The direct link between defective OXPHOS activity in cancer cell metabolism and apoptosis having been demonstrated in published work, we have focused in this manuscript on the metabolic responses to doxorubicin drug treatment of aggressive (African-American, E006AA) and more responsive (Caucasian-American, LNCaP) PCa cells using our FLIM-FRET assay, hypothesizing that FLIM-based markers should show the correction of impaired OXPHOS. The goals were to identify (i) the molecular events associated with the changes in mitochondrial energy metabolism/OXPHOS activity upon doxorubicin treatment, and (ii) the early predictors of drug response by following the lifetimes (τ_1_, τ_2_, τ_m_) and their relative fractions (a_1_%, a_2_%) of NAD(P)H, FAD, Trp; NAD(P)H-a_2_%/FAD-a_1_% FLIM-based redox ratio, NAD(P)H-Trp FRET interactions (E%), E% vs NAD(P)H-a_2_%/FAD-a_1_% median correlation along-with biochemically quantifying the generation of ROS and caspase-3 activity. The FLIM-based redox ratio results of this study clearly demonstrate lower metabolic activity before doxorubicin treatment and early molecular changes after treatment associated with increased OXPHOS after 5 time-points each with a 15 min interval up to 60 min, preceding the effector caspase-3 activation and apoptosis. Based on our FLIM method for quantitative analysis of mitochondrial energy metabolism, we differentiate between the (1) Pre-Apoptotic, (2) Responsive and (3) Slow responder PCa cells. Previous studies have utilized NAD(P)H and FAD lifetime changes for the evaluation of metabolic variations in cancer^34–36^. For the first time, we demonstrate that along with NAD(P)H and FAD lifetime changes, Trp-quenching due to Trp-NAD(P)H FRET interactions, and E% vs NAD(P)H-a_2_%/FAD-a_1_% median correlation are sensitive parameters in predicting the drug response of PCa cells.

## Results

### Induction of mitochondrial OXPHOS activity upon doxorubicin treatment

**Figure 1:**
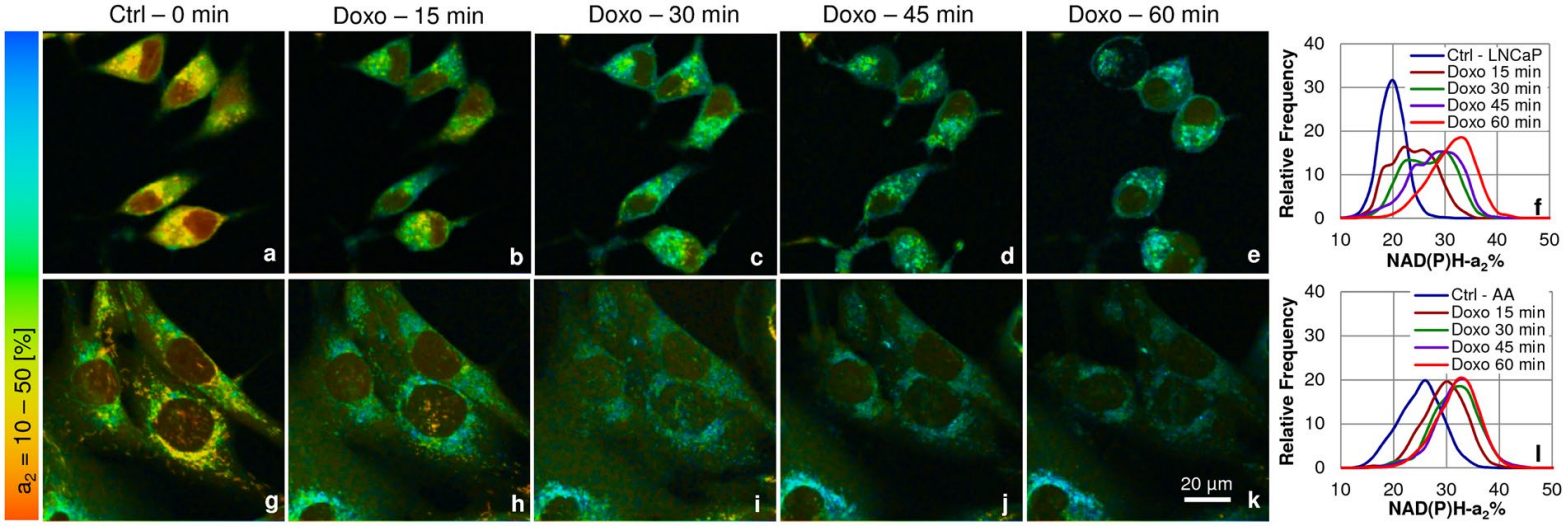
Increase in the enzyme-bound fraction of NAD(P)H-a_2_% upon doxorubicin treatment in PCa cells. Upper panel (**a**–**f**) LNCaP and lower panel (**g**–**l**) E006AA (AA) cells. PCa cells: LNCaPs (n = 26) with 4400–4890 ROIs, and E006AA cells (n = 28) with 5585–6372 ROIs, each ROI of 2 × 2 pixels, were analyzed for 5 time points (0, 15, 30, 45 & 60 min). The representative (**a**–**e** and **g**–**k**) color coded images and (**f** and **l**) the histograms showed increase in the enzyme-bound fraction of NAD(P)H-a_2_% with time (0–60 min) upon treatment with 1 µM doxorubicin when compared with untreated 0 min time point control (Ctrl). Greater magnitude of response was seen in LNCaP vs E006AA cells (60% vs 23%) from 0 to 60 min.

**Figure 2:**
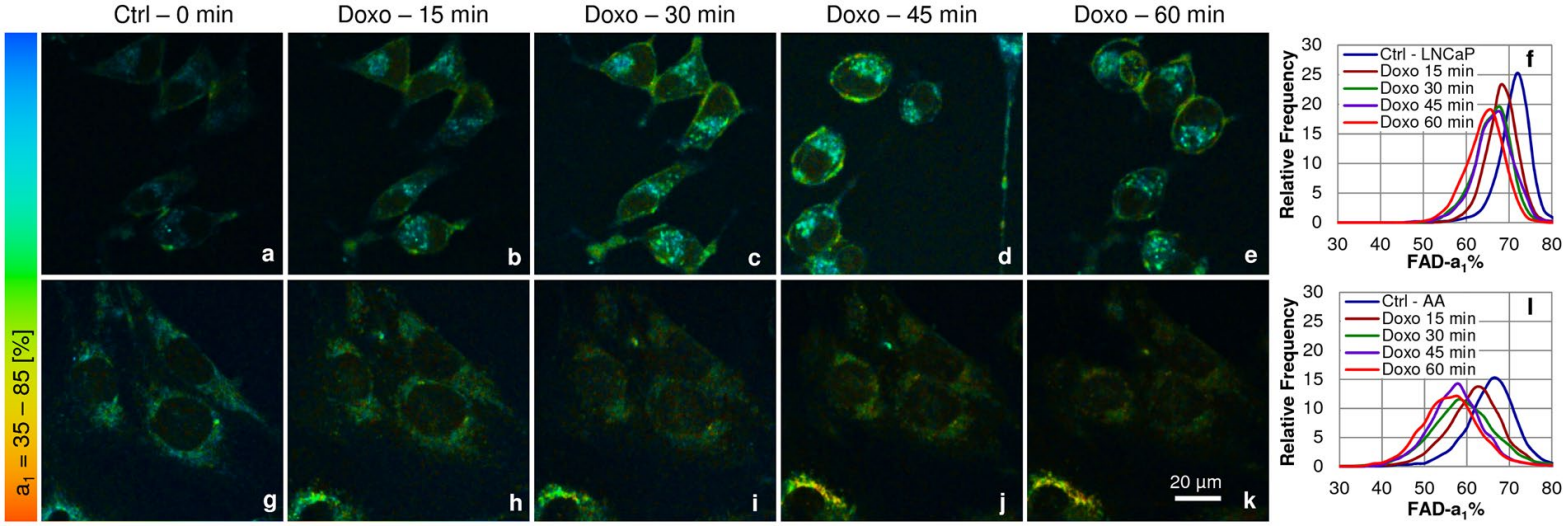
Decrease in the enzyme-bound fraction of FAD-a_1_% upon doxorubicin treatment in PCa cells. Upper panel (**a**–**f**) LNCaP and lower panel (**g**–**l**) E006AA (AA) cells. PCa cells: LNCaPs (n = 26) with 4400–4890 ROIs, and E006AA cells (n = 28) with 5585–6372 ROIs, each ROI of 2 × 2 pixels, were analyzed for 5 time points (0, 15, 30, 45 & 60 min). The representative (**a**–**e** and **g**–**k**) color coded images and (**f** and **l**) the histograms showed decrease in the enzyme-bound fraction of FAD-a_1_% with time (0–60 min) upon treatment with 1 µM doxorubicin when compared with untreated 0 min time point control (Ctrl).

Metabolic changes in the mitochondria were investigated in LNCaP and E006AA PCa cells before and after doxorubicin treatment using our 3-channel FLIM-FRET assay. FLIM images were acquired at time zero as control and upon treatment with doxorubicin at 15 min intervals for 60 min. Doxorubicin-treated LNCaP cells showed a more impaired cellular morphology like cell shrinkage and some blebbing (Fig. 1a–e), whereas E006AA cells (Fig. 1g–k) morphologically appeared to be less affected by the cytotoxic effects of doxorubicin during the treatment time course. The mean lifetimes of NAD(P)H at the mitochondrial loci increased over the time course (0–60 min) in both cell lines upon treatment with doxorubicin (Fig. S2). Mitochondrial FAD mean lifetimes showed an increase in E006AA cells over the same time course, but no appreciable change in LNCaP cells (Fig. S3). Before analyzing the FLIM-based redox state, the frequency distribution of the two individual enzyme-bound fractions of NAD(P)H-a_2_% (Fig. 1f and l) and FAD-a_1_% (Fig. 2f and l) were examined.

The mitochondrial OXPHOS activity utilizes NAD(P)H and produces FAD. In other words, there is more enzyme-bound NAD(P)H-a_2_% and less enzyme-bound FAD-a_1_% fractions. In agreement with this, NAD(P)H-a_2_% increased over the time course while FAD-a_1_% decreased, exhibiting an increase in OXPHOS activity with doxorubicin treatment over time, suggesting normalization of an impaired mitochondrial function and opening the apoptotic pathway, mentioned in the introduction. The peak change of NAD(P)H-a_2_% from 0 min control to 60 min after doxorubicin treatment, was an 60% increase in LNCaP and 23% increase in E006AA cells (Fig. 1f and l); FAD-a_1_% decreased by 8.3% in LNCaP and 9.1% in E006AA cells (Fig. 2f and l).

**Figure 3:**
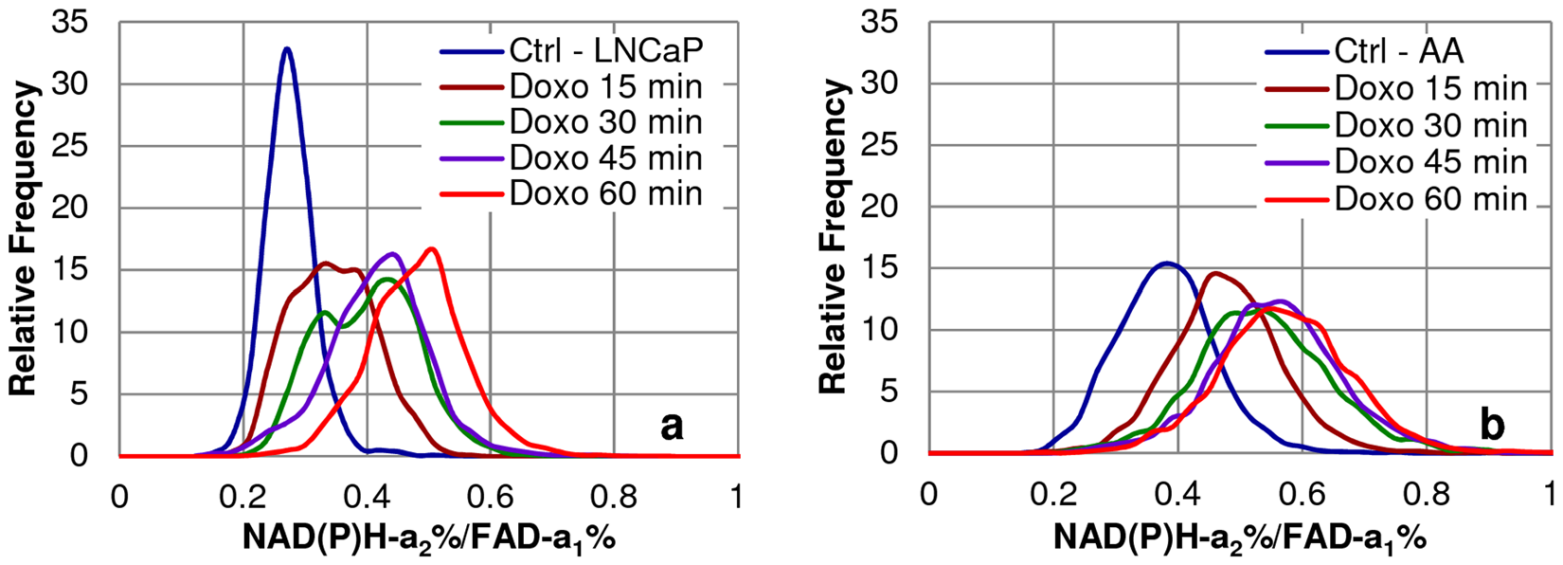
Increase in mitochondrial NAD(P)H-a_2_/FAD-a_1_ Redox Ratio upon doxorubicin treatment in PCa cells. Mitochondrial OXPHOS activity uses NAD(P)H (more enzyme bound-a_2_%) and produces FAD (less enzyme bound-a_1_%). The histograms showed increase in mitochondrial NAD(P)H-a_2_/FAD-a_1_ Redox Ratio in (**a**) LNCaP and (**b**) E006AA (AA) cells for the same ROIs (mentioned above) with time (0–60 min) upon treatment with 1 µM doxorubicin compared with 0 min untreated control (Ctrl). The data suggests that there is increased mitochondrial OXPHOS activity with doxorubicin treatment. Greater magnitude of response was seen in LNCaP vs E006AA cells (89% vs 38%) from 0 to 60 min.

In line with above observation, an increase of the FLIM redox ratio was observed with doxorubicin treatment over time in both PCa cell lines (Fig. 3), mainly driven by the increase of NAD(P)H-a_2_% and less so by the decrease of FAD-a_1_% (Figs 1f and 1, 2f and l). The peak change of FLIM-based redox ratio from 0 min control to 60 min after doxorubicin treatment was an 89% increase in LNCaP (Fig. 3a) and a 38% increase in E006AA (Fig. 3b). Increase in all these parameters indicates overall increase in OXPHOS activity after doxorubicin treatment. Hence, these FLIM parameters can be used as early predicators of drug-response.

### Increased E% in the Trp-NAD(P)H FRET interaction correlates with increased metabolic activity upon doxorubicin treatment

**Figure 4:**
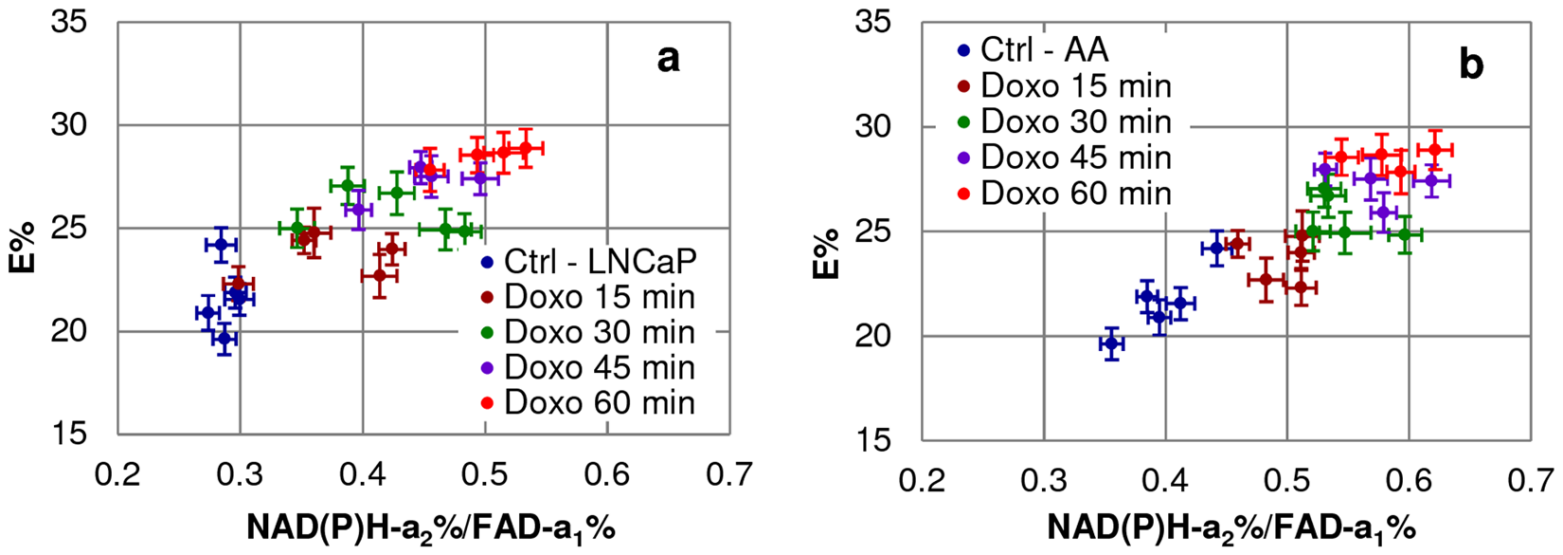
Increase in E% with increase in mitochondrial NAD(P)H-a_2_/FAD-a_1_ Redox Ratio upon doxorubicin treatment in PCa cells. Correlation plots showed increase in median E% w.r.t. median NAD(P)H-a_2_/FAD-a_1_ redox ratio from control (Ctrl) to doxorubicin treatment groups with time (0–60 min) in both (**a**) LNCaP and (**b**) E006AA (AA) cells which suggests increased interaction of Trp (residues in proteins/enzymes) with NAD(P)H with increased mitochondrial OXPHOS activity.

We have previously demonstrated that increased Trp-NAD(P)H FRET interaction and increased E% upon stimulation with glucose in cancer cells as an indicator of increased metabolic activity^13, 37^. This E% increase correlates with the increase in the enzyme-bound fraction of NAD(P)H (a_2_%). In the current study, we have extended the analysis to correlate with the FLIM-based redox ratio, showing that E% increased with rising redox states. Figure 4 tracks median E% by Field-of-view (FoV) covering >5000 Regions of Interest (ROIs) and correlates E% with the NAD(P)H-a_2_%/FAD-a_1_% redox ratio. E% increased concurrently with the increase in the FLIM-based redox ratio in both PCa cells suggesting that E% - NAD(P)H-a_2_%/FAD-a_1_% median correlation can be used to predict the drug-response in PCa cells.

### Increase in the mitochondrial oxidative stress upon doxorubicin treatment

**Figure 5:**
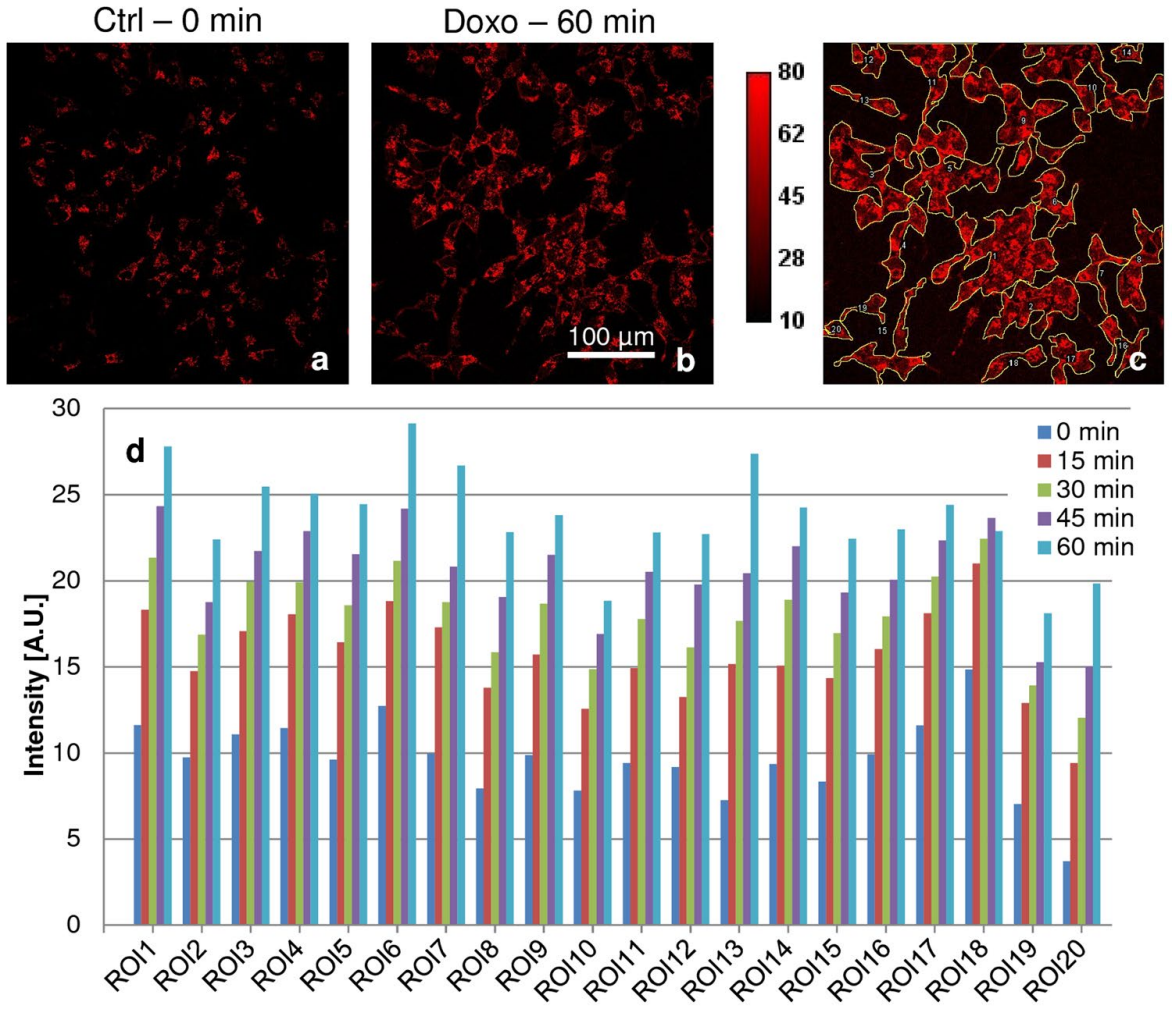
Increase in mitochondrial ROS upon doxorubicin treatment. LNCaP cells were labeled with MitoSox Red. After imaging of the control cells for 0 min time point, 1 µM doxorubicin was added on stage and proceeded with time lapse imaging at 15 min intervals up to 60 min. Representative images of cells (n = ~100) (**a**) 0 min control (Ctrl), (**b**) increased ROS at 60 min doxorubicin treatment, (**c**) auto-contrasted image with clusters of adjoining cells (n = ~100) grouped together to represent individual ROIs as numbered, (**d**) were quantified and showed a 1.5-to 5.4-fold increase in ROS over time after doxorubicin treatment.

Increase in the production of ROS induces oxidative stress which results in oxidization of cellular macromolecules, inhibits protein function and promotes cell death^38^. Increased ROS is shown to be involved in doxorubicin induced apoptosis in cancer cells^29^. Usually, such studies have used 24–48 hr time points to assess induction of ROS and apoptosis. The current study’s objective *inter alia* was to identify early predictors of drug response and we therefore, correlated the observed changes in the NAD(P)H, FAD and Trp lifetimes and their relative fractions with the induction of ROS over 0–60 min period of doxorubicin treatment. MitoSOX Red is a selective live cell dye for detecting superoxide – the predominant ROS in the mitochondria. We observed increase in ROS as early as 15 min of doxorubicin treatment, which gradually increased over time (Fig. 5). Representative images of untreated 0 min control (Fig. 5a) and doxorubicin treated for 60 min (Fig. 5b) are shown. Clusters of adjoining cells (n = ~100) were grouped together to represent individual ROIs as numbered in Fig. 5c. Each ROI was quantified from 0–60 min and shows a 1.5- to 5.4-fold increase in ROS over time after doxorubicin treatment (Fig. 5d). Therefore, generation of ROS, which indicates beginning of oxidative cellular damage, correlates with the increase in the FLIM parameters in the early phase of doxorubicin treatment.

### Induction of apoptosis upon doxorubicin treatment

**Figure 6:**
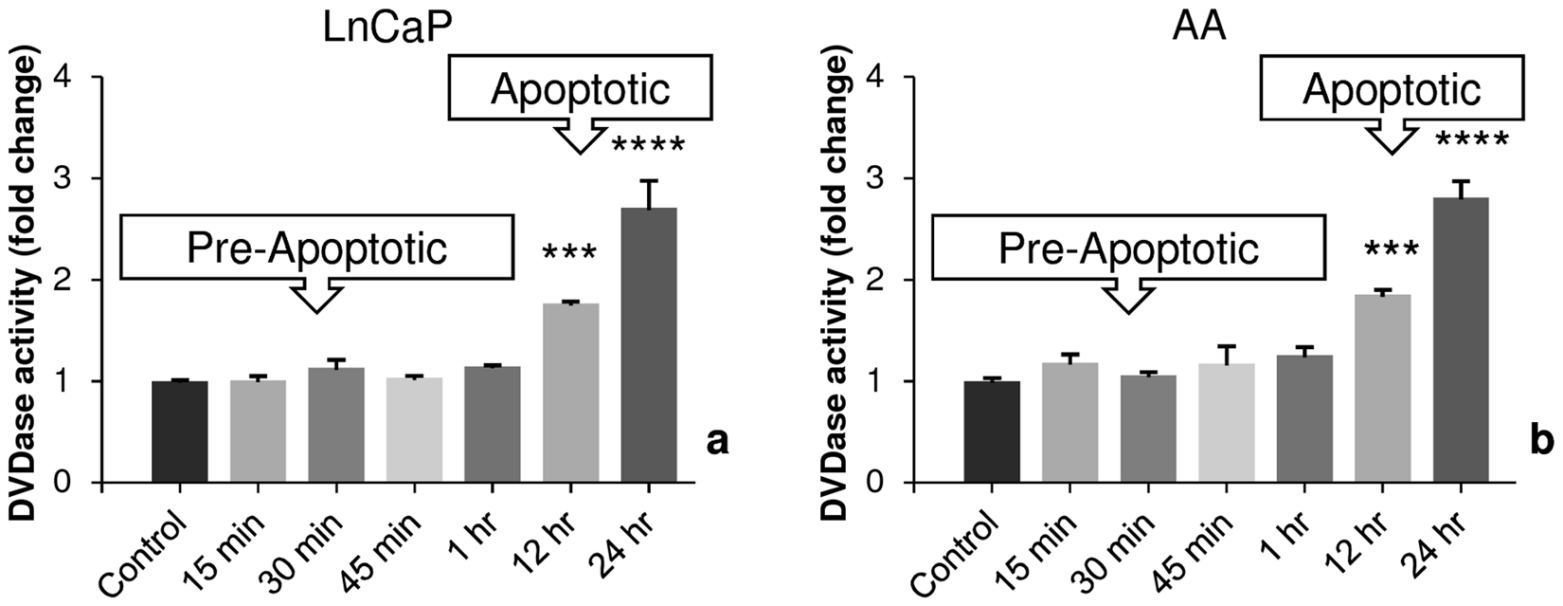
Increase in caspase-3 activity with time upon doxorubicin treatment in PCa cells. (a) LNCaP and (**b**) E006AA (AA) PCa cells were treated with 1 µM doxorubicin for different time points and assessed for the effector caspase-3 activity by DEVDase assay. There was no increase in caspase-3 activity in earlier time points 0 min–1 hr (Pre-Apoptotic Phase), which increased significantly ***p < 0.001 and ****p < 0.0001 at 12 hr and 24 hr (Apoptotic Phase) with doxorubicin treatment as compared to control group.

Doxorubicin has been shown to induce apoptosis in various types of cancer cells^32, 33^. However, the early changes in the mitochondrial NAD(P)H, FAD and Trp lifetimes prior to apoptosis upon doxorubicin treatment were not defined. Apoptosis is a long process with a cascade of upstream and downstream events, ultimately causing the activation of the effector caspase-3 which executes the final steps of apoptosis^39^. Apoptosis was therefore, analyzed by measuring caspase-3 activity by a DEVDase assay in both PCa cell lines. We have extended the time period to the point of detecting caspase activity to show that our shorter FLIM assay actually predicts the later apoptotic changes. No caspase-3 activity was detected at earlier time points (0–60 min) of doxorubicin treatment. At 12 hr and 24 hr time points post doxorubicin treatment, caspase-3 activity in both LNCaPs and E006AA cells occurred (Fig. 6). Based on the −/+ activation of effector caspase-3, we have categorized these phases as “Pre-Apoptotic” and “Apoptotic or Responsive” phases, respectively.

### Indicators of doxorubicin drug-response

**Figure 7:**
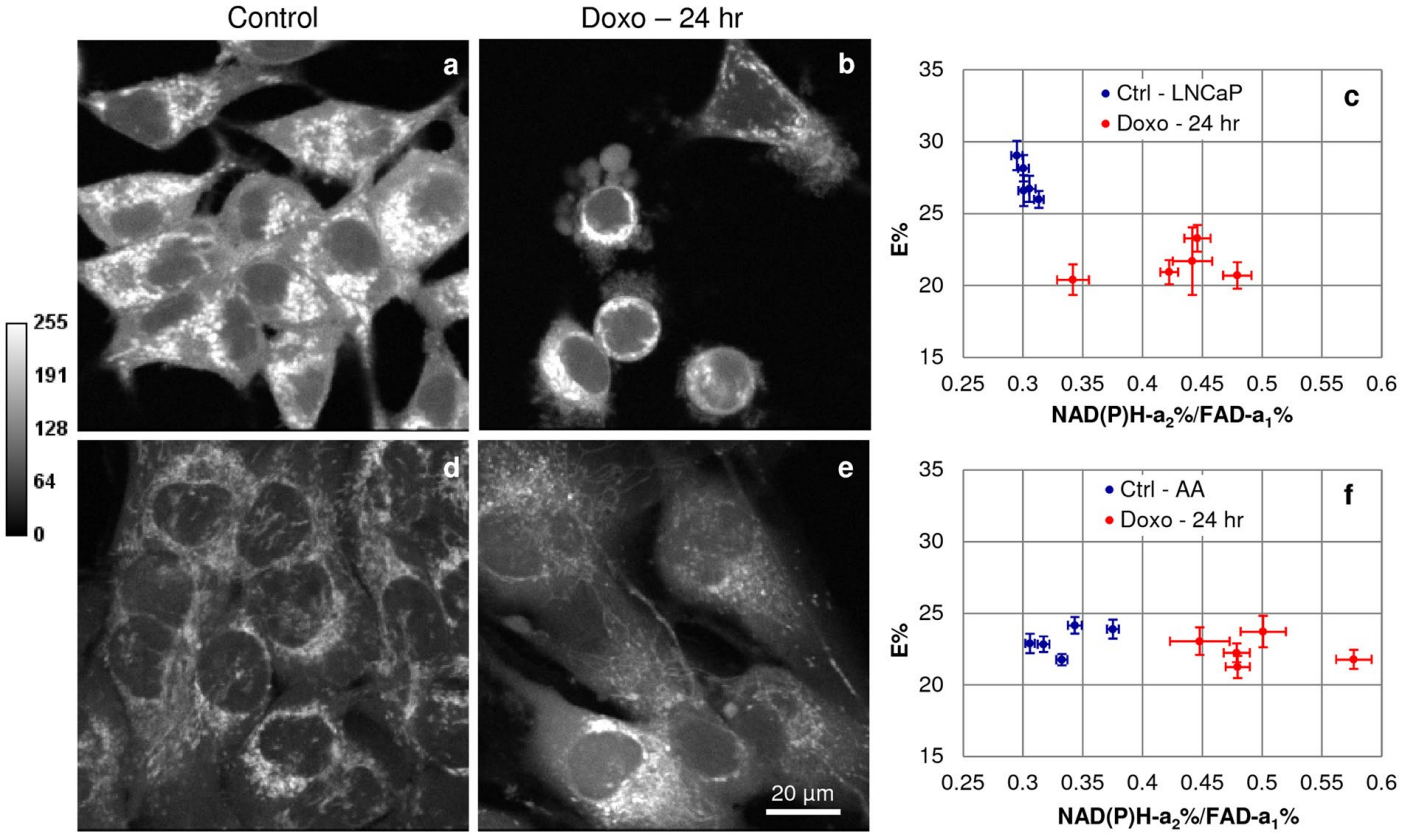
Median correlation of E% vs NAD(P)H-a_2_%/FAD-a_1_% an indicator of doxorubicin responsiveness. Upper panel (**a**–**c**) LNCaP and lower panel (**d**–**f**) E006AA (AA) PCa cells. Representative (**a**,**b** and **d**,**e**) NAD(P)H photon images show apoptotic cell morphology in (**b**) LNCaPs-doxorubicin “Responsive” cells as compared to the (**a**) LNCaP control cells; whereas the (**d**) E006AA control and (**e**) E006AA-doxorubicin “Slow Responders” look morphologically similar. The median correlation of E% vs NAD(P)H-a_2_%/FAD-a_1_% showed (**c**) decrease in E% in LNCaPs-doxorubicin “Responsive” cells and (**f**) no change in E% in E006AA-doxorubicin “Slow Responders” with increase in NAD(P)H-a_2_%/FAD-a_1_% ratio.

Metabolic changes in the “apoptotic phase” after 24 hr doxorubicin treatment in LNCaP vs E006AA cells showed increases in the mean lifetime of NAD(P)H and FAD (not shown), increases in NAD(P)H-a_2_% (33.3% vs 36.4%, Fig. S4a and d), decreases in FAD-a_1_% (10.3% vs 25.0%, Fig. S4b and e) and increases in NAD(P)H-a_2_%/FAD-a_1_% FLIM-based redox ratio (30% vs 60%, Fig. S4c and f). LNCaP cells exhibited morphological signs of apoptosis accompanied by cell shrinkage, condensed cytoplasmic organelles and extensive plasma membrane blebbing (Fig. 7b) and were termed as doxorubicin “Responsive” cells. In contrast, these morphological abnormalities were not so prominent in E006AA cells and therefore were termed as doxorubicin “Slow Responders” (Fig. 7e). Correlation of medians of E% and NAD(P)H-a_2_%/FAD-a_1_% showed decrease in E% in LNCaPs doxorubicin “Responsive” cells (Fig. 7c). Interestingly, we saw no change in E% in E006AA doxorubicin “Slow Responders” as compared to the control with the increase in redox ratio (Fig. 7f), which suggests that these cells had a slower response to treatment.

Applying the categorization of “Pre-Apoptotic” (0–60 min of treatment) and “Responsive”, “Slow Responders” (after 24 hr treatment) to NAD(P)H-τ_m_, NAD(P)H-a_2_%, FAD-a_1_%, NAD(P)H-a_2_%/FAD-a_1_% FLIM-based redox ratio, similar trends were observed across these 3 categories (Table 1). Only the median correlation of E% with NAD(P)H-a_2_%/FAD-a_1_% parameter was able to differentiate these 3 categories and depict the heterogeneity in their doxorubicin drug responses showing that E% increases in the “Pre-Apoptotic” phase, decreases in the “Responsive” cells and no change in the “Slow Responders”.

**Table 1:** FLIM-FRET parameters predictors of doxorubicin drug response in PCa cell lines.

FLIM parameters/Molecular Targets	Molecular Changes in LNCaP & E006AA Doxorubicin Rx in “Pre-Apoptotic” Phase (0–60 min)	Molecular changes in LNCaPs Cells Doxorubicin “Responsive” (~24 hr)	Molecular changes in E006AA Cells Doxorubicin “Slow Responders” (~24 hr)
NAD(P)H-τ_m_	Increases	Increases	Increases
NAD(P)H-a_2_%	Increases	Increases	Increases
FAD-a_1_%	Decreases	Decreases	Decreases
NAD(P)H-a_2_%/FAD-a_1_% FLIM Redox Ratio	Increases	Increases	Increases
E% vs NAD(P)H-a_2_%/FAD-a_1_% Correlation	Increases	Decreases	No Change

FLIM E% calculation is commonly based on Trp-τ_m_ [(τ_1_ *a_1_%) + (τ_2_ *a_2_%)] which contains, both, the fractions of bound and free Trp and relating more closely to intensity-based E% calculations, which cannot differentiate between bound and free moieties. Extending the analysis to Trp-τ_m_ and Trp photons counts in these 3 categories tracks the Trp quenching by FRET events occurring in categories. The “Pre-Apoptotic” phase showed decrease in Trp-τ_m_ and Trp photons counts in both PCa cells which reflects increased Trp quenching (see Fig. S5a–d) with increase in metabolic activity; LNCaP-“Responsive” cells showed increase in Trp-τ_m_ and increasing trends in Trp photon counts (p = 0.054) which reflects less Trp quenching (see Fig. S5e–f) in the apoptotic cells, whereas the E006AA “Slow Responder” cells showed no appreciable change in Trp-τ_m_ or in Trp photon counts (p = 0.1) from control (see Fig. S5g–h). Our results, demonstrates that Trp-quenching and median E% correlation with NAD(P)H-a_2_%/FAD-a_1_% can be used as sensitive indicators to track a drug response in cancer in pre-apoptotic, apoptotic and slow responder cells, which is one of the most sought after goals in treatment to be able to identify and track a treatment response in heterogeneous cancer cell populations.

## Discussion

In the current study, we have investigated the metabolic response of a potent anti-cancer drug doxorubicin on two PCa cell lines African American (E006AA or AA) and Caucasian American (LNCaP) by FLIM of NAD(P)H, FAD and Trp in the mitochondria. Differences in all applied parameters are observed between CA and AA PCa cell lines, the poorer response to treatment in more aggressive AA PCa’s being attributed to the lower mtDNA^28^. Mitochondrial dysfunction and defective OXPHOS activity are recognized as the cause of this disparity^27^. It is interesting to note, that inhibition of the OXPHOS complex I causes a reduction in the release of mitochondrial cytochrome c, which contributes to the inhibition of caspase activation and apoptosis^29^. Although, the exact role of OXPHOS in regulating apoptosis is not clearly understood, our previous findings suggest that restoration of mitochondrial function induces ROS production leading to permeabilization of mitochondrial membrane and apoptosis induction^29, 40^. Therefore, correction of the impaired mitochondrial OXPHOS activity and subsequent restoration of the apoptosis induction are promising strategies in cancer treatment. Since molecular changes precede morphological or phenotypic changes, this investigation identifies the molecular changes as the early predictors of drug response using our FLIM-FRET assay.

Our results in these two cell lines match those of patient-based data^28, 41–43^, except that our assay could be applied at an earlier stage of PCa for diagnosis and treatment. The assay identifies morphological changes, level of cytotoxicity and magnitude of metabolic response upon doxorubicin treatment in LNCaP vs E006AA cells in the “Pre-Apoptotic” phase with the peak change of 60% vs 23% increase in NAD(P)H-a_2_% (see Fig. 1f and l) and 8.3% vs 9.1% decrease in FAD-a_1_% fractions (Fig. 2f and l) and 88.88% vs 38.46% increase in the NAD(P)H-a_2_%/FAD-a_1_% (Fig. 3a and b) FLIM-based redox from 0 min to 60 min of treatment. These metabolic changes were accompanied with the increased Trp-NAD(P)H interaction resulting in increased E% which correlated with NAD(P)H-a_2_%/FAD-a_1_% redox ratio (Fig. 4). Also, there was increased ROS generation (Fig. 5) followed by induction of apoptosis (Fig. 6).

Both LNCaP and E006AA cells showed induction of apoptosis at 12 hr and 24 hr. We compared changes in the FLIM-FRET parameters in the “Pre-Apoptotic” (0–60 min) and apoptotic (~24 hr) phases to delineate the differences and interpret the doxorubicin drug-induced metabolic response in these phases. Interestingly, two groups emerged: LNCaP doxorubicin “Responsive” and E006AA doxorubicin “Slow Responder” PCa cells (Table 1). The LNCaP doxorubicin- “Responsive” cells showed morphological signs of apoptosis (Fig. 7b) whereas in the E006AA doxorubicin “Slow Responder” cells such morphological changes were not observed (Fig. 7e). However, at this stage, it is difficult to conclude that whether the E006AA cells were resistant to apoptosis or whether they had a slower response to treatment; which may be influenced by variation in experimental culture conditions, drug dose and duration of treatment; real-time mapping of apoptosis and FLIM-FRET metabolic responses to drug treatment within the same cell, at the same time would help to elucidate and correlate these molecular changes with apoptosis. In a recent study, FLIM changes and ratiometric caspase biosensor activity in an antibiotic induced apoptosis has been reported^35^. Similar to ours, studies have shown increase in OXPHOS with staurosporine antibiotic induced apoptosis in colon cancer and HeLa cells^35, 44^.

The current study provides spatial and temporal resolution of “Responsive” and “Slow Responder” cells and demonstrates Trp-quenching and median E% correlation with NAD(P)H-a_2_%/FAD-a_1_% as sensitive indicators to track the doxorubicin drug response in PCa cells. Using the E% and NAD(P)H-a_2_%/FAD-a_1_% median correlation we were able to categorize PCa cells into “Pre-Apoptotic”, “Responsive” and “ Slow Responders”, one of the most sought after goals in cancer treatment and follow up. Future direction of this study would be real-time mapping of the identified FLIM-FRET parameters with the generation of ROS and apoptosis in *in vitro* spheroid and organoid cultures and later in PCa mouse model to assess heterogeneity and track the treatment response.

Another potential application of the FLIM assay relates to elevated mitochondrial glutaminolysis in PCa compared to normal tissue^45, 46^. The enzyme Glutaminase (GLS) coverts glutamine to glutamate; glutamate is subsequently converted to α-ketoglutarate by glutamate dehydrogenase using NAD(P)^+^ as an co-enzyme and producing non-enzyme bound (free) NAD(P)H, contributing to the free NAD(P)H pool, while mitochondrial OXPHOS contributes to the enzyme-bound NAD(P)H fraction^1, 15^. PCa’s dysregulation in glycolysis, glutaminolysis and OXPHOS pathways compared to normal cause levels of free and enzyme-bound NAD(P)H fractions to vary. Using the FLIM assay to measure the altered levels of the NAD(P)H fractions, we could differentiate normal from PCa cells that is dependent on mitochondrial glutaminolytic pathway.

In conclusion, this study shows FLIM & FRET has the capability to detect earlier molecular changes associated with the correction in mitochondrial OXPHOS activity before the onset of apoptosis with doxorubicin treatment in PCa cells. Hence, our investigation provides molecular basis that induction of cell death by correction of impaired mitochondrial OXPHOS activity are promising strategies in cancer treatment. We observed these changes as early as 15 min of doxorubicin treatment accompanied with the increase in ROS. Also, this study reports for the first time, Trp-quenching due to Trp-NAD(P)H interactions (FRET), and E% vs NAD(P)H-a_2_%/FAD-a_1_% median correlation are sensitive parameters in tracking the drug response of PCa cells. Therefore, these parameters can be used as early predictors of drug response and in assessing the efficacy of anti-cancer drug treatment.

## Materials and Methods

### Cell Culture

Different PCa cell lines from African-American (E006AA) and Caucasian-American (LNCaP) origins have been used in this study. The E006AA (or AA) cells were maintained in high-glucose Dulbecco’s Modified Eagle Medium (Life Technologies) supplemented with 10% cosmic calf serum (Hyclone), 1% Penicillin-Streptomycin (Life Technologies), and 4 mM Sodium Pyruvate (Life Technologies). The LNCaP cells were maintained in RPMI 1640 (Life Technologies) supplemented with 10% cosmic calf serum (Hyclone) and 1% Penicillin-Streptomycin (Life Technologies). All cells were maintained in the cell culture incubator, at 37 °C with 5% CO_2_.

### FLIM Instrumentation, Processing and Analysis

Our 3-channel FLIM imaging system consists of a Zeiss LSM 780 confocal/multiphoton (MP) laser scanning system coupled to the Zeiss inverted epi-fluorescence microscope, which is controlled with the ZEN software (Carl Zeiss, Inc). Multiphoton excitation of Trp, NAD(P)H and FAD was achieved by using an ultrafast (150 fs) tunable Ti:sapphire laser (680–1060 nm), operating at 80 MHz repetition rate (Chameleon Vision II, Coherent, Inc.). To excite the NAD(P)H and Trp we used 740 nm (NAD(P)H: 2-photon ecx, ET480/40 em; Trp: 3-photon exc, HQ360/40 em)^13^ and 890 nm for FAD (2-photon exc, 540/40 em). The fluorescence decay per pixel was measured using 3-channel SPC-150 TCSPC board (Becker & Hickl, GmbH) where the SPCM software was used to acquire the FLIM data (v. 8.91). Details on the methodology of our FLIM set up can be found elsewhere^9, 47^. A Zeiss 40 × 1.3NA oil, (EC Plan-Neuofluar, UV transmission is 60% at 340 nm) objective lens was used to focus the light on the sample and collect the emission for 60 s. The average power at the specimen plane (7 mW) and the acquisition time was chosen to reduce any photodamage to the cells.

After simultaneous acquisition of FLIM images for Trp, NAD(P)H and FAD, the florescence lifetime images were fitted for 2-components using SPCImage software (v. 5.5, Becker & Hickl). Number of parameters was generated including photon images, τ_1_, τ_2_, a_1_%, a_2_%, and χ^2^ for each pixel of each channel. Since, NAD(P)H signal matches with the mitochondrial morphology (Fig. S1), the mitochondrial Regions of Interest (ROI)s were thresholded by 2 × 2 pixels/ROI using the NAD(P)H photon image. The generated mitochondrial ROIs were used for further FLIM data analysis. The exported results from multiple samples and FOVs were further analyzed as described elsewhere^9, 47^.

### Fluorescence Lifetime Imaging Microscopy and Doxorubicin Treatment

For imaging, LNCaP and E006AA PCa cells were plated onto 25 mm round #1.5 glass coverslips (Thermo Scientific), in their recommended growth medium and grown to 70–80% confluence. Fluorescence lifetime imaging was acquired in phenol-free or in FluoroBrite-DMEM (Thermo Fisher Scientific) growth medium with the microscope-heated stage maintained at 37 °C and under the flow of humidified blood-gas mixture (5% CO_2_). After imaging of the control FoVs, 1 µM doxorubicin was added in the growth medium on stage and FLIM data was again acquired at 15 min interval up to 60 min.

### Quantification of the Optical Redox Ratio

For the optical redox ratio calculation, we have used a FLIM-based method which uses NAD(P)H-a_2_%/FAD-a_1_% redox pairs^9^. Our preferred FLIM-based NAD(P)H-a_2_%/FAD-a_1_% ratio avoids potential intensity-related artefacts, such as photo-bleaching or fluctuations of illumination levels.

### Quantification of Mitochondrial ROS

LNCaP cells plated on glass coverslips were incubated with 500 nM MitoSOX Red reagent (Molecular Probes, Invitrogen) in HBSS Buffer with 5 mM Glucose for 45 min at 37 °C in the cell culture incubator. Live cell imaging was implemented in growth medium with heated stage maintained at 37 °C and under humidified gas flow on the Leica TCS SP5X using 40x water immersion, NA 1.1, excitation with white light laser 510 nm at 59% laser intensity and emission was collected for 550–650 nm range with 750 V PMT gain. After imaging of the control FoVs, 1 µM doxorubicin was added in the growth medium on stage and time lapse imaging was done at 15 min interval for 60 min. Images were exported as “tiff” and Region of Interests (ROIs) for groups of cells were drawn and quantified (n = ~100 cells) in Fiji.

### Quantification of the Caspase Activity

PCa cells were treated with 1 µM doxorubicin for different time points as described in Figure legends. Treated Cells (LNCaP & E006AA) were washed with PBS, and the whole cell lysates were made in the sample lysis buffer (50 mM HEPES, 1% Triton X-100, 0.1% CHAPS, 1 mM dithiothreitol (DTT), and 0.1 mM EDTA). 40 μg of protein was added to a reaction mixture containing 30 μM fluorogenic peptide substrates, DEVD-AFC, 50 mM of HEPES, pH 7.4, 10% glycerol, 0.1% CHAPS, 100 mM NaCl, 1 mM EDTA, and 10 mM DTT, and incubated at 37 °C for 90 min. Fluorescence from 7-amino-4-trifluoromethylcoumarin (AFC) was monitored in a spectrofluorimeter (Biotek Synergy 2) using excitation wavelength 400/30 nm and emission wavelength 508/20 nm. The results are presented as fold activation over the control^48^.

### Statistical Analysis

Results are expressed as mean ± SEM. Differences between groups were analyzed for statistical significance using a Student’s t-test and one-way ANOVA analysis wherever applicable. The threshold for the significance level or p-value of the test was 0.05.

### Data Availability

All data generated or analyzed in this study are included in this manuscript (and in the Supplementary Information).

## Electronic supplementary material

Supplementary Info
