# Supplementary material for: Investigation of Mitochondrial Metabolic Response to Doxorubicin in Prostate Cancer Cells: An NADH, FAD and Tryptophan FLIM Assay
Source: Sci Rep. 2017 Sep 5;7:10451. doi: 10.1038/s41598-017-10856-3 (PMC5585313; doi:10.1038/s41598-017-10856-3)
Supplement: Supplementary file 1 — Supplementary Info [file 41598_2017_10856_MOESM1_ESM.pdf]

Supplementary Information

Investigation of Mitochondrial Metabolic Response to Doxorubicin in Prostate Cancer Cells: An NADH, FAD and Tryptophan FLIM Assay

Shagufta Rehman Alam[a], Horst Wallrabe[a], Zdenek Svindrych[a], Ajay K. Chaudhary[b] Kathryn G. Christopher[a], Dhyan Chandra[b], and Ammasi Periasamy[a,c*]

[a]The W.M. Keck Center for Cellular Imaging, Physical and Life Sciences Building, [c]Departments of Biology and Biomedical Engineering, University of Virginia, 90 Geldard Dr., Charlottesville, Virginia, USA 22904.

[b]Roswell Park Cancer Institute, BLSC-Centre for Genetics and Pharmacology, Department of Pharmacology and Therapeutics, Elm & Carlton Streets, Buffalo, New York, USA14263.

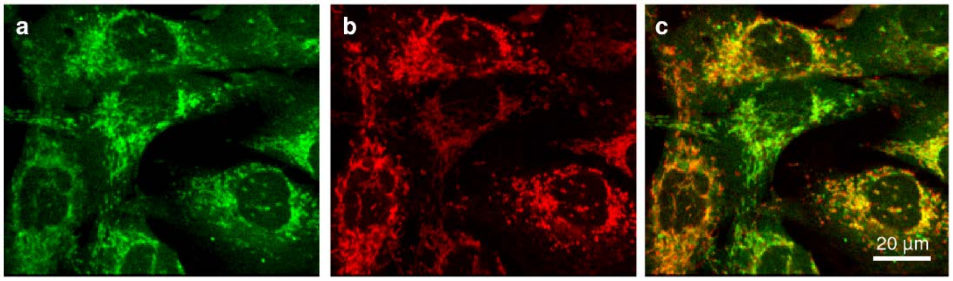

Figure S1: NAD(P)H auto-fluorescence signal matches with mitochondrial staining. Representative photon image of (a) NAD(P)H; (b) mitochondria labeled with 100 nM MitoTracker Red FM and (c) superimposed image showing NAD(P)H signal matches with mitochondrial staining in E006AA PCa cells.

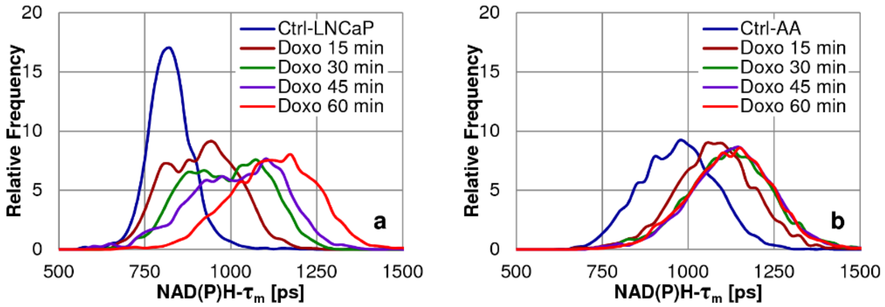

Figure S2: Increase in the mean lifetime of NAD(P)H in (a) LNCaP and (b) E006AA (AA) cells upon doxorubicin treatment over the time (0 – 60 min) as compared with the control (Ctrl).

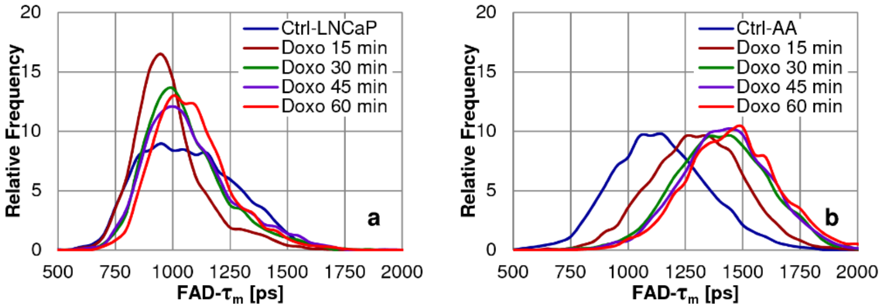

Figure S3: FAD mean lifetime in (a) LNCaP-no appreciable change and (b) increase in E006AA (AA) cells upon doxorubicin treatment over the time (0 – 60 min) as compared with the control (Ctrl).

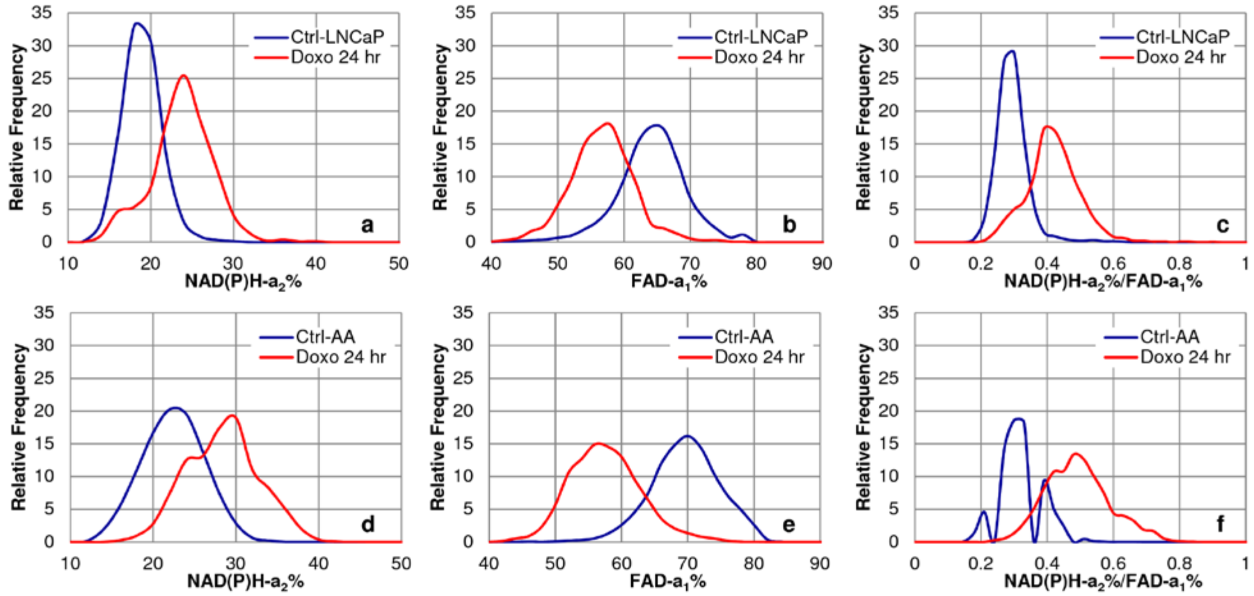

Figure S4: Doxorubicin induced metabolic changes at 24 hr treatment**.** Upper panel (a-c) LNCaP and lower panel (d-f) E006AA (AA) PCa cells. Increase in (a,d) NAD(P)H-a_2%, decrease in (b,e) FAD-a_1% and increase in (c,f) NAD(P)H-a_2%/FAD-a_1% lifetime based redox ratio as compared to the control (Ctrl) was seen in both PCa cell lines.

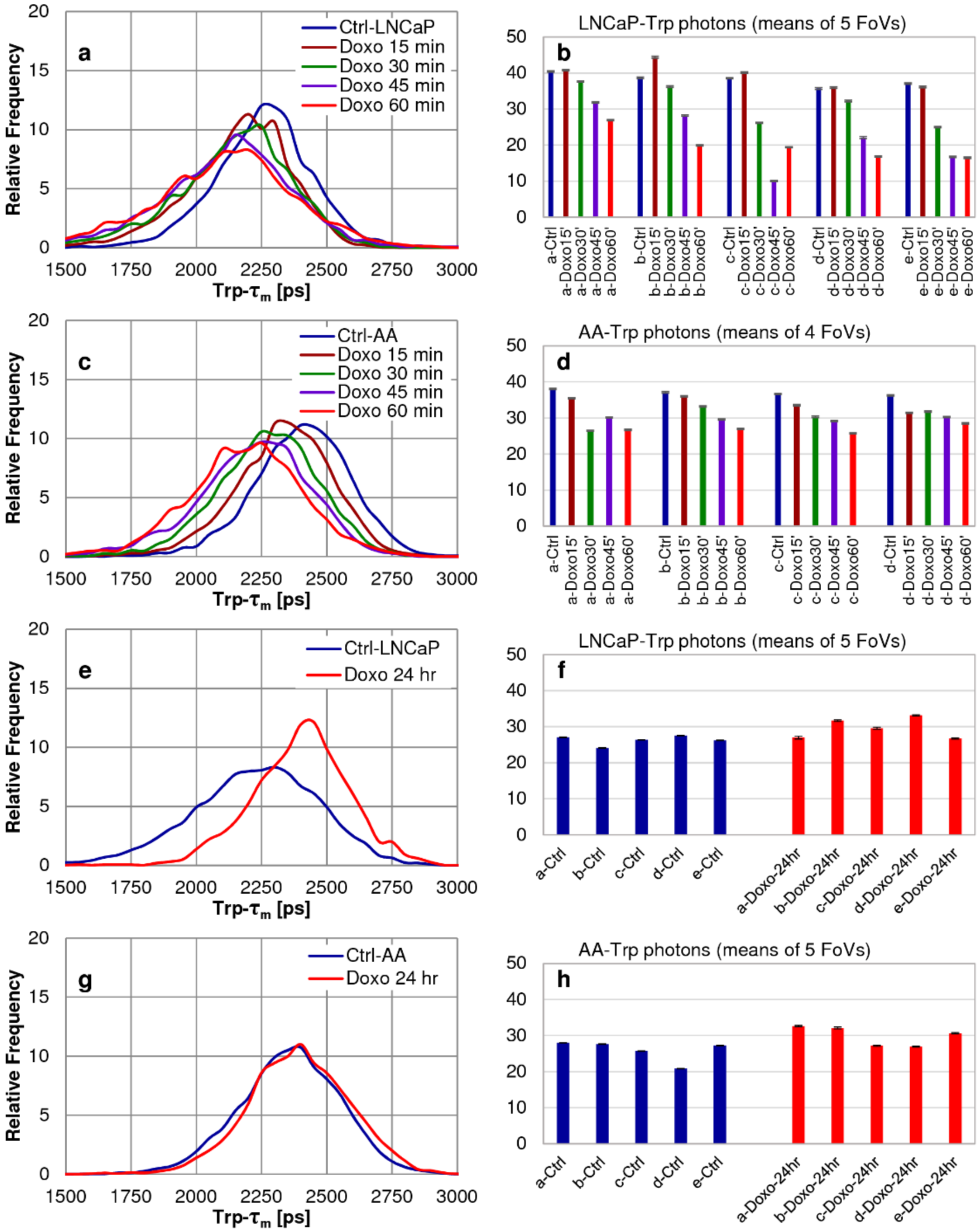

Figure S5: **Trp-quenching a measure of PCa cells's responsiveness to doxorubicin treatment**. In the pre-apoptotic phase: both the (a,c) histograms of mean fluorescence lifetime and the (b,d) bar charts for photon counts of Trp showed decrease compared with the untreated 0 min time point control (Ctrl) in LNCaP (a-b) and (c-d) E006AA (AA) PCa cells which suggests increased quenching of Trp fluorescence with increased metabolic activity. In (e-f) LNCaP doxorubicin "Responsive"/apoptotic cells there was increase in (e) Trp-τ_m and increasing trends in (f) Trp-photon counts ($p = 0.054$) whereas in (g-h) E006AA doxorubicin "Slow Responder" cells no change in (g) Trp-τ_m or in (h) Trp-photons ($p = 0.1$) when compared to untreated controls.
